# Risk of Depression and Suicidality among Diabetic Patients: A Systematic Review and Meta-Analysis

**DOI:** 10.3390/jcm7110445

**Published:** 2018-11-16

**Authors:** Rasha Elamoshy, Yelena Bird, Lilian Thorpe, John Moraros

**Affiliations:** 1 School of Public Health, University of Saskatchewan, Saskatoon, SK S7N 2Z4, Canada; rasha.elamoshy@usask.ca (R.E.); yelena.bird@usask.ca (Y.B.); 2 Community Health and Epidemiology, University of Saskatchewan, Saskatoon, SK S7N 2Z4, Canada; lilian.thorpe@usask.ca

**Keywords:** diabetes, depression, suicide, suicidal ideation, suicidal attempts, suicidal death

## Abstract

The purpose of this study is to conduct a systematic review and meta-analysis to evaluate the risk of depression and suicidality among diabetic patients. Methods: Medline, PubMed, EMBASE, Cochrane library, and Psych INFO were searched for studies published from 2008 onwards. Meta-analysis was conducted to estimate the pooled effect size. Sources of heterogeneity were investigated by subgroup analysis and meta-regression. Results: In total, 5750 articles were identified and of those, 17 studies on suicidality and 36 on depression were included in this study. Our analysis suggests a positive relationship between diabetes and depression (cohort studies odds ratio (OR) 1.49, 95% confidence interval (CI): 1.36–1.64 and cross-sectional studies OR 2.04, 95% CI, 1.73–2.42). Pooled OR values for suicidal ideation, attempted suicide, and completed suicide were 1.89 (95% CI: 1.36–2.63), 1.45 (95% CI: 1.07–1.96), and 1.85 (95% CI: 0.97–3.52), respectively. All findings were statistically significant except for completed suicide. Conclusions: The increased risk of depression and suicidality in diabetic patients highlights the importance of integrating the evaluation and treatment of depression with diabetes management in primary healthcare settings. Further research in this area is needed.

## 1. Introduction

Diabetes is considered one of the largest global epidemics and constitutes a public health emergency in many countries [1]. The global prevalence of diabetes among adults has nearly doubled in the last couple of decades, rising from 4.7% in 1980 to 8.5% in 2014, resulting in approximately 1.6 million deaths annually [2]. Diabetes is psychologically demanding given the chronic and large burden placed on diabetics for the self-management of their disease. Diabetic patients face several psychological challenges as a result of their illness, which may include: adherence to medical treatment and lifestyle modifications, need for continued monitoring for glycemic control, concerns for complications and disabilities, interference of symptoms with daily activities, and psychosocial difficulties at personal and interpersonal levels [3], all of which may eventually lead and interconnect to depression and in some cases suicide.

Depression is a common mental illness that negatively impacts productivity and quality of life [4]. It is reported that patients with depression die 5–10 years earlier than those without depression [5]. Evidence suggests that the co-morbidity of depression and diabetes is relatively common [6,7,8]. A previous meta-analysis estimated the prevalence of depression to be twice as high among diabetics compared to the general population [6]. Other systematic reviews corroborated these findings and demonstrated a significantly higher risk for diabetic patients to develop depression compared to non-diabetics [7,8].

A recent study found no genetic link to account for the positive association between diabetes and depression [9]. However, certain epigenetic factors (low socioeconomic status, irregular sleep patterns, lack of physical activity, and poor diet) may play an important role in activating common physiological pathways (stress and inflammation systems) that promote and reinforce diabetes and depression [10]. Chronic stress activates the hypothalamus–pituitary–adrenal axis (HPA axis) and the sympathetic nervous system (SNS), leading to increased production of cortisol and adrenalin/noradrenalin, respectively [11]. Chronic levels of increased cortisol and prolonged secretion of adrenalin/noradrenalin may promote diabetes (insulin resistance) [12] and depression (dopamine dysfunction) [13] or both (disturbance of neurogenesis in the hippocampus) [14]. Furthermore, chronic stress increases the production of pro-inflammatory cytokines that can lead to diabetes (by disturbing the normal functioning of the pancreatic β-cells) [15] and depression (by disrupting neuroendocrine function) [16].

The co-morbidity between diabetes and depression is associated with poorer prognosis and higher medical expenditures than either condition alone [17]. This is an expected finding as depression significantly impacts diabetic patients leading to poor self-care, non-adherence to medical treatment, reduced quality of life, higher rates of morbidity and mortality, and consequently, increased healthcare costs [18,19]. Additionally, depression among diabetic patients tends to last longer and has higher recurrence rates compared to non-diabetics [20]. Consequently, identification of depression among diabetic patients is critically important to mitigate these negative personal consequences and realize cost-savings in healthcare.

Suicide was responsible for more than 800,000 deaths per year in 2015 and is the second leading cause of death among those aged 15 to 29 years old worldwide [21]. Depressive symptoms may increase the risk of diabetic patients displaying suicidality [22], which may include: suicidal ideation, attempted suicide, and completed suicide. Although the association between diabetes and depression has been previously examined, the association with suicidality remains unclear and the evidence is limited and at times, conflicting [23,24,25].

Despite the documented interconnections, there is a significant gap in the literature regarding the systematic assessment of depression and suicidality in patients with diabetes. To the best of our knowledge, the present study is one of the first meta-analyses of its kind and adds value to our understanding on this important research topic. The purpose of this study was to conduct a systematic synthesis and meta-analysis of the evidence in order to: (1) assess the risk of depression (including clinical depression, depressive symptoms, and use of antidepressants); (2) determine the prevalence of suicidality; and (3) evaluate the risk of suicidality among diabetic patients.

## 2. Experimental Section

### 2.1. Data Sources and Search Strategies

A systematic literature search was undertaken using five relevant databases: PubMed, Medline, EMBASE, Psych INFO, and Cochrane Library. Search strategies focused on three major domains: (1) diabetes; (2) depression or suicidality; and (3) quantitative outcome measures.

### 2.2. Eligibility Criteria and Study Selection

In our study, eligible articles were required to: (1) be published in the English language in peer-reviewed journals since 2008, and be available in full text; (2) assess patients with type 1 and/or type 2 diabetes mellitus who either self-reported a physician’s diagnosis of diabetes, were prescribed anti-diabetic medications, or were participants in studies using laboratory-based assessments; and (3) evaluate depressive disorders, or use of antidepressants or depressive symptoms based on validated standardized questionnaires. Studies were also selected by scanning the reference lists of previous systematic reviews on the topic. Articles had to include both exposed and non-exposed groups of participants and provide sufficient data to calculate odds ratio for depression among diabetics. Eligible articles were identified through title and abstract screening, followed by full text review. Two reviewers independently evaluated studies for relevance in a standardized manner. Non-agreement was resolved by discussion and adjudication.

### 2.3. Data Extraction

Data extraction strategies were developed and pilot-tested on 20 randomly selected included studies and then modified accordingly. Information extracted in duplicate from studies included: author, publication year, country, study design, follow-up time (for cohort studies), settings, total number, age and sex of study participants, method of diabetes evaluation, method of depression or suicidality assessment, reported effect measure with 95% confidence interval, and covariates for adjusted effect measures. Unadjusted odds ratios and 95% confidence intervals were calculated using the number of events in the exposed and non-exposed groups.

### 2.4. Risk of Bias

Two reviewers independently assessed the validity of eligible studies by using a modified version of the Newcastle Ottawa Scale (NOS) [26]. The NOS assesses representativeness of the study sample, comparability between respondents and non-respondents, ascertainment of depression or suicidality, and thoroughness of the reported descriptive statistics. Studies were rated as having a low, moderate, or high risk of bias.

### 2.5. Data Analysis

Statistical analysis was performed using the Comprehensive Meta-analysis software-version 3 (CMA-3) [27]. For depression, pooled odds ratios (both adjusted and unadjusted) were the effect measure of interest. While for suicidality, both odds ratios and pooled prevalence among diabetics were the focus of our analysis. As heterogeneity was likely to exist, a random effect model was used to calculate pooled estimates, which allows for estimating both within and between studies variation. We examined heterogeneity using the Cochran’s Q heterogeneity test and I^2^ as a measure for inconsistency [28]. Influential analysis was conducted for the effect of each study on the pooled estimate by reassessing estimates after removal of one study at the time. To visually assess for publication bias, the funnel plot method was used. The Egger’s regression intercept method was also used to confirm and statistically test for the bias observed in the funnel plot [29]. When publication bias was detected, a Duval and Tweedie trim and fill method was used to calculate the adjusted effect size [30]. All analysis used a 5% level of significance (*α* = 0.05).

## 3. Results

### 3.1. Study Selection

**Figure 1:**
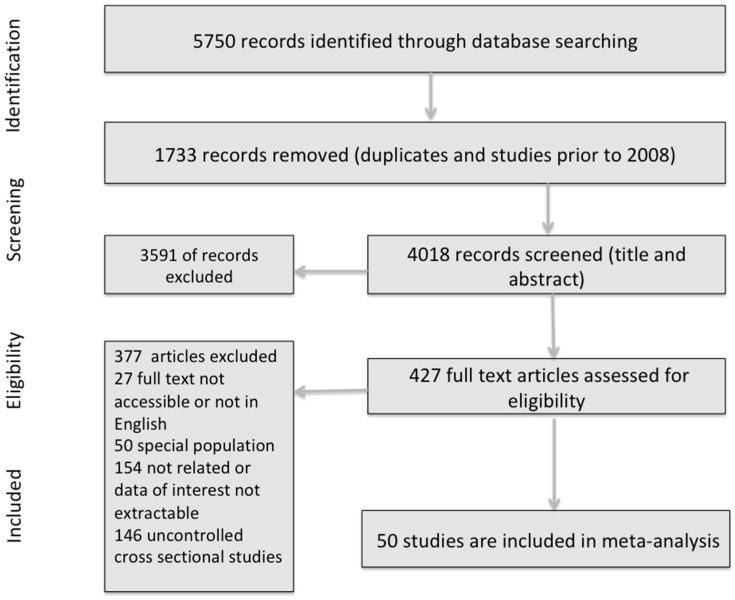
Preferred Reporting Items for Systematic Reviews and Meta-Analyses (PRISMA) flow chart for study selection process.

The PRISMA flow chart depicting the study selection is shown in Figure 1. A comprehensive search of the literature yielded a total of 5750 articles from which 427 articles qualified for full text screening and 355 were excluded due to one or more of the following conditions: (1) their full text was not available; (2) they were not written in English; (3) they focused on special populations; and/or (4) reported inadequate data or mixed outcomes. In total, 50 studies were analyzed, with 33 reporting data only on depression, 14 only on suicidality, and three studies reporting data on both depression and suicidality among diabetic patients [31,32,33].

### 3.2. Study Characteristics

#### 3.2.1. Depression

Depression studies were stratified and examined on the bases of their study design. Table 1 and Table 2 include summary of the characteristics of depression studies included in the review.

(1) Cohort Studies

There were 12 cohort studies included for depression. One study [34] reported two datasets for patients with prevalent and incident diabetes, leaving a total of 13 datasets for analysis. Three studies [35,36,37] used incident prescription of antidepressants as a proxy for depression diagnosis, five studies [34,38,39,40,41] used clinical diagnostic criteria, and three [42,43,44] relied on questionnaires. One study used both questionnaires and prescription of antidepressants [42]. The follow-up period ranged from two [34] to 15 years [36]. Most studies were from Europe or North America, while only two studies were from Asia [38,39]. Ascertainment of diabetes was made by physician diagnosis (International Classification of Diseases (ICD) code) [34,36,38,39,40,43], use of anti-diabetic medications [35,37,43], self-reported diagnosis of diabetes [44,45], and/or laboratory assessment [42,43].

(2) Cross-Sectional Studies

There were 23 cross-sectional studies and one case-control study [46] on depression and diabetes. Additionally, three of these studies reported data on suicidality [31,32,33]. Depression was evaluated by: (1) questionnaires; (2) clinical diagnostic criteria (nine studies) [31,32,46,47,48,49,50,51,52]; and (3) utilization of antidepressants (one study) [53]. Two studies [47,54] used both depressive disorders and symptoms but for the analysis, we used only results for depressive disorders.

#### 3.2.2. Suicidality

Suicidality was stratified and examined on the bases of outcome. Table 3 and Table 4 include summary of the characteristics of suicidality studies included in the review

(1) Completed Suicide

Seven studies reported data on completed suicide (all cohort). Suicidal death was confirmed by either International Classification of Diseases (ICD) codes or examination of death certificate.

(2) Suicidal Attempts

Five studies assessed suicidal attempts and self-harm (one cohort, one nested case-control and three cross-sectional). Attempted suicide was evaluated based on either ICD codes or self-reported information.

(3) Suicidal Ideation

Nine cross-sectional studies examined suicidal ideation. Three of them evaluated type 1 diabetics, one study evaluated type 2 diabetic patients, who were on insulin [32], while the remaining studies included both type 1 and type 2 patients. Two studies had minors as participants (adolescents) [25,73]. All studies used self-reported information to evaluate for the presence of suicidal ideation either as a response to a single question, or as a part of depression screening questionnaires, or during a standardized interview.

### 3.3. Main Meta-Analysis Results

#### 3.3.1. Depression

Depression results are reported as odds ratios (adjusted and unadjusted) by study design. The overall OR based on all depression studies (cohort and cross-sectional) was 1.79 (95% CI: 1.62–1.99), with significant heterogeneity (*I*^2^ = 98.09%, *Q* = 9.66, and *p*-value < 0.001).

(1) Cohort Studies

**Figure 2:**
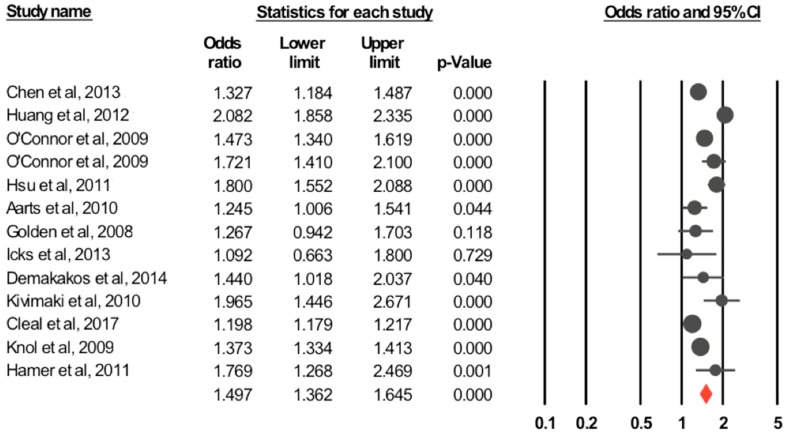
Forest plot for the association between depression and diabetes from cohort studies. Estimates are in the center of the box and lines represent 95% confidence intervals (CI). Diamond shows the pooled odds ratio size and its 95% CI.

The pooled unadjusted odds ratio for the association between diabetes and depression calculated based on all 13 studies, using a random effect model was 1.49 (95% CI: 1.36–1.64, *p*-value < 0.001). Forest plot of the OR and 95% CI for the random effect model are shown in Figure 2. There was significant heterogeneity between studies (*I*^2^ = 94.08%, *Q* = 203.12, *p*-value < 0.001). The pooled adjusted effect estimate was calculated based on five studies [35,42,43,44,45] with an OR = 1.48 (95% CI: 1.16–1.88) and *p*-value = 0.001. The test for heterogeneity was not significant (*I*^2^ = 54.2%, *Q* = 8.73, *p*-value = 0.068).

(2) Cross-Sectional Studies

Unadjusted odds ratio for depression among diabetics, calculated based on cross-sectional studies was 2.04 (95% CI: 1.73–2.42) *p*-value < 0.001. However, there was a significant heterogeneity (*I*^2^ = 93.79%, *Q* = 370.7, *p*-value < 0.001). The adjusted odds ratio from cross-sectional studies was 1.67 (95% CI: 1.47–1.90) *p*-value < 0.001, with significant heterogeneity (*I*^2^ = 74.16%, *Q* = 58.05, *p*-value < 0.001).

#### 3.3.2. Suicidality

Suicidality results are reported as prevalence and odds ratios (adjusted and unadjusted) stratified by outcome.

(1) Completed Suicide

Overall prevalence was 0.3% (95% CI: 0.1–0.7%). Based on the five included studies [55,56,57,58,59], the unadjusted pooled odds ratio was 1.85 (95% CI: 0.97–3.52, *p*-value = 0.061) (Figure 2), with significant heterogeneity (*I*^2^ = 94.79%, *Q* = 76.88, *p*-value < 0.001). Adjusted OR (based on three studies) was 1.39 (95% CI: 0.82–2.35, *p*-value= 0.225) (*I*^2^ = 81.66%, *Q* = 10.90, *p*-value = 0.004).

(2) Suicidal Attempts

The pooled prevalence of attempted suicide was 2.7% (95% CI: 0.9–7.8%). Three studies reported unadjusted odds ratios. The calculated unadjusted pooled odds ratio was 1.45 (95% CI: 1.07–1.96, *p*-value = 0.017) (Figure 2), with significant heterogeneity (*I*^2^ = 53.43%, *Q* = 4.29, *p*-value = 0.117). Adjusted odds ratio based on two studies [31,69] was 1.33 (95% CI: 1.09–1.62, *p*-value = 0.005) (*I*^2^ = 0%, *Q* = 0.23, *p*-value = 0.635).

(3) Suicidal Ideation

The prevalence of suicidal ideation, among diabetics was 16.2% (95% CI: 8.5–28.5%). Unadjusted pooled odds ratio (based on six studies) was 1.89 (95% CI: 1.36–2.63, *p*-value < 0.001) (Figure 2), with significant heterogeneity (*I*^2^ = 94.09%, *Q* = 84.65, *p*-value < 0.0001). Adjusted odds ratio (based on four studies) was 1.49 (95% CI: 1.14–1.96, *p*-value = 0.004) (*I*^2^ = 80.37%, *Q* = 15.28, *p*-value = 0.002).

### 3.4. Subgroup Analysis and Meta-Regression

#### 3.4.1. Depression

(1) Cohort Studies

In the six studies that evaluated depression among diabetics using clinical diagnostic criteria, the pooled OR was 1.59 (95% CI: 1.35–1.86) and the test of heterogeneity was significant (*I*^2^ = 88.09%, *Q* = 41.98, *p*-value < 0.001). In the four studies that evaluated depression among diabetics using questionnaires, the pooled OR was 1.41 (95% CI: 1.17–1.69), while the test of heterogeneity was not significant (*I*^2^ = 9.24%, *Q* = 3.30, *p*-value = 0.340). For the three studies that used antidepressants, the pooled OR was 1.36 (95% CI: 1.19–1.54) and test of heterogeneity was significant (*I*^2^ = 97.32%, *Q* = 74.52, *p*-value 0.001). Studies were stratified on the basis of assessing patients with incident diabetes OR = 1.49 (95% CI: 1.33–1.69) and prevalent diabetes OR = 1.47 (95% CI: 1.25–1.72). Other confounders (level of adjustment, comparison group, risk of bias, and geographical location) did not significantly impact the effect estimate. Results for meta-regression are included in Table 5.

(2) Cross-Sectional Studies

In evaluating depression, the same pattern observed for cohort studies was maintained in cross-sectional studies. In examining the differences between groups, depressive disorders (OR = 2.24, 95% CI: 1.46–3.45), depressive symptoms (OR = 1.47, 95% CI: 1.37–1.57), and antidepressants (OR = 1.89, 95% CI: 1.86–1.92) were not statistically significant (*p*-value = 0.738).

#### 3.4.2. Suicidality

**Figure 3:**
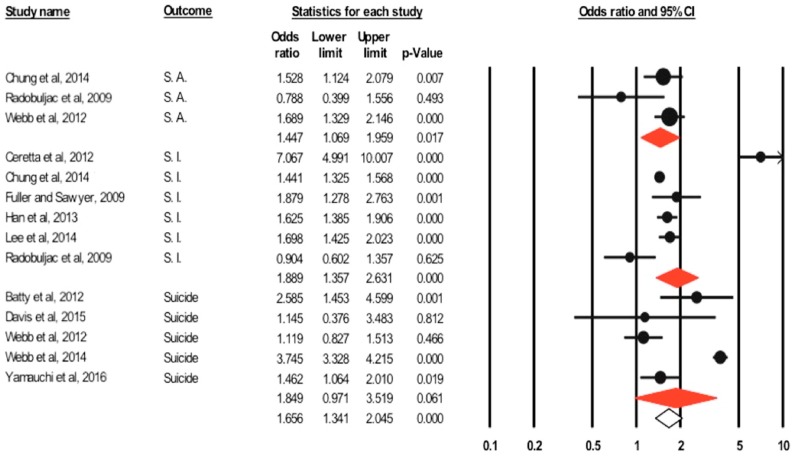
Forest plot of unadjusted odds ratio for suicidality and diabetes. SA: suicidal attempts, SI: suicidal ideation. Red diamonds represent pooled odds ratio for each outcome, while white diamond represent the overall odds ratio for the three outcomes.

The results for suicidality subgroup analysis are included in Table 6, Figure 3. Due to the limited number of studies assessing attempted suicide, no subgroup analysis was conducted.

### 3.5. Influential Analysis and Publication Bias

**Figure 4:**
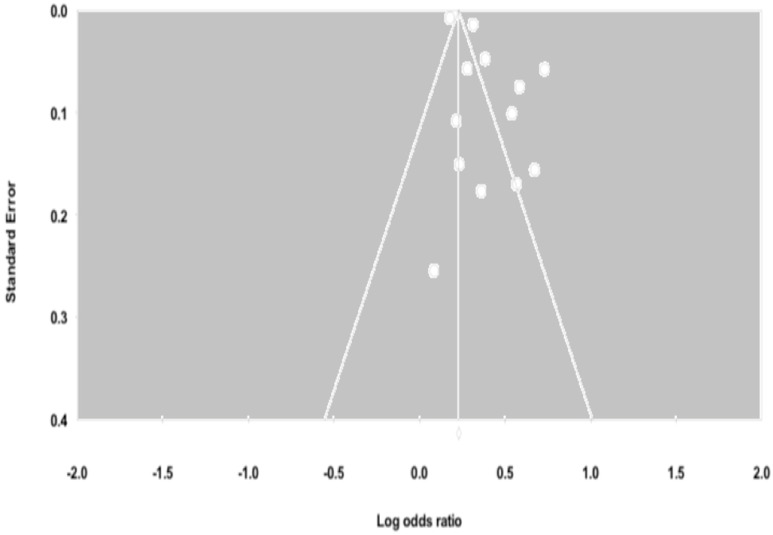
Funnel plots of the meta-analysis of published studies. Each point represents the log odds ratio and the standard error for a single study. The triangle represents the region where 95% of the data points would lie in the absence of a publication bias. The vertical line represents the average log odds ratio found in the meta-analysis.

Influential analysis revealed that no single study had a substantial influence on either the adjusted or unadjusted effect estimates. There was evidence of publication bias for depression among cohort studies based on the inspection of the funnel plot (Figure 4) and Egger’s test (*p*-value = 0.027). To account for this bias, a Duval and Tweedie test was used and reported an adjusted OR 1.25 (95% CI: 1.15–1.37). There was no evidence of publication bias for depression among cross-sectional studies (*p*-value = 0.721) and suicidality studies (*p*-value = 0.860).

## 4. Discussion

To our knowledge, this study is one of the first meta-analyses that reports the prevalence of suicidality among diabetic patients and assesses the association between diabetes, depression, and suicidality using data from observational studies. Our results show that diabetic patients are more likely to have depression, experience suicidal ideations and attempt suicide compared to non-diabetic patients. The findings of this study help highlight diabetics as a high-risk group for depression and suicidality.

In regards to depression, based on 13 cohort studies and 23 cross-sectional studies, our results suggest that there is a significant association between depression and diabetes. It is interesting to note that this association maintained its strength even after adjusting for potential confounders. Previous systematic reviews have shown that patients diagnosed with diabetes are at higher risk for depression [7,78]. Similarly, our analysis of cohort studies corroborates the existing evidence [78] and suggests a directionality, whereby diabetes may play a causal role in the development of depression. However, the psychological burden of diabetes may lead to but does not fully account for the increased risk of depression [3]. Other potential physiological contributors include: activation of the HPA axis and SNS [11,79], chronic inflammation [80], and cerebral vascular changes induced by diabetes [81]. Additionally, some common medications used for the treatment of diabetes have been linked to a higher risk of depression [82,83]. 

In our study, the calculated odds ratio for cross-sectional studies (assessing prevalence of depression) was higher than the one in cohort studies (assessing incidence of depression), corroborating the results reported in a previous meta-analysis [6]. This finding may be explained in part by: (1) the longer duration of depression among diabetic patients, and (2) the potential bidirectional association between diabetes and depression. The longer duration of depression among diabetic patients may be due to the increased likelihood to experience treatment resistant and recurrent forms of depression [20]. This leads to a buildup of chronic cases. The potential bidirectional association between diabetes and depression has also been examined in the literature [42,84]. Depression has been linked to the development of type 2 diabetes with the use of certain antidepressants, which are known to have clinical effects on glucose homeostasis and weight gain [85]. Additionally, depression may have a negative effect on a patient’s lifestyle choices including physical activity, leading to an increased risk to develop diabetes [86]. Thus, it is unsurprising that the risk of prevalent depression was higher than that of incident depression among diabetic patients in our study.

Suicidality among diabetic patients has not been fully elucidated. Our study examined the prevalence and risk of suicidality among diabetic patients in order to address the existing gap in the literature. In the general population, a study involving 17 countries found the lifetime prevalence of suicidal ideation and attempts to be at 9.2% and 2.7%, respectively [87]. By comparison, our study found that the prevalence of suicidal ideation among patients with diabetes was much higher, at a reported 16.2% (95% CI: 8.5–28.5%), while the rate of attempted suicide was similar, at 2.7% (95% CI: 0.9–7.8%).

Depression is one of the leading risk factors for suicidality [22,88]. In our study, diabetic patients were found to be twice as likely to experience suicidal ideations compared to non-diabetics. When examining attempted suicide, diabetics were also significantly at higher risk compared to non-diabetics. These findings are concerning and help highlight the vulnerability of diabetic patients possibly progressing from suicidal ideation to attempted suicide. Despite the increased risk of suicidal ideation and attempts, diabetic patients did not experience a significant risk of suicidal death (OR = 1.85, 95% CI: 0.97–3.52, *p*-value = 0.061). This could be attributed to several reasons connected to suicide, including: (1) stigmatization; (2) misclassification; (3) low occurrence; (4) limited details and number of studies; and (5) lack of research into the distinct predictive factors for suicidal death.

Stigma related to suicide is a major barrier in accurately reporting and tracking of suicidal deaths [89]. The high level of stigmatization in many countries, where suicide is considered to be immoral and illegal, might lead to underreporting of suicide as a cause of death [90]. This in turn would negatively impact accuracy of suicide rates reported in large-scale epidemiological studies [90]. Misclassification of suicidal death due to insulin overdoses as accidental death or death due to natural causes is another possibility that need to be further evaluated. Studying suicide is also statistically challenging because of the relative low occurrence of the event and the need for large samples to obtain reliable estimates [91]. Additionally, in the present systematic review, the limited number of studies assessing suicidal attempts lacked some key details. For example, there was dearth of information assessing the seriousness of the attempt (i.e., extent of hospital care required afterward or whether the attempt resulted in permanent disabilities). Furthermore, it has been suggested in the literature that suicidal ideations and behaviors might have different predictors than completed suicide [92,93]. Therefore, we cannot fully rely on suicidal ideations to understand completed suicide. Factors that may play a role in moving from one condition to another remain unclear for diabetic patients and need to be carefully investigated. 

### 4.1. Strengths and Limitations

This study has several strengths. It provides an up-to-date literature review, which includes both depression and suicidality as outcomes of interest among diabetic patients. It provides evidence that can be used as a reference point for future research focusing on specific diabetic sub-populations. Finally, it takes into consideration several important factors (i.e., method of depression evaluation, type of diabetes, whether diabetes was incident or prevalent, and geographical location) in the analysis of our data, which adds to the robustness of this study.

However, there are also several limitations we need to consider. First, there are few studies assessing suicidality, especially attempted suicide. Second, there was a marked heterogeneity among the included studies. Third, we included studies that used prescription of antidepressants as a proxy for depression. This method is quite controversial especially among diabetic patients since antidepressants are commonly used for symptomatic treatment of diabetic neuropathic pain, which may falsely increase reported estimates. However, our subgroup analysis showed that differences in depression evaluation methods did not significantly impact our results. Fourth, confounding bias could not be entirely eliminated. Fifth, association of diabetes and suicidal attempts and suicidal ideation do not imply causation. Finally, most studies included in our systematic review were from developed countries and therefore, the results of our analysis need to be interpreted with caution, as they may not be generalizable to the developing world.

### 4.2. Implications for Future Research and Clinical Practice

There are significant gaps and opportunities for research in this important public health topic. Further research is warranted to: (1) examine the factors, mechanisms and transitional triggers implicated in the association between diabetes, depression and suicidality; (2) assess the role and impact of different diabetes management strategies on the patient’s risk of depression and suicidality; and (3) evaluate the cultural and ethnic differences as they relate to diabetes, depression and suicidality. Clinicians should be aware and receive cross-training in order to be better prepared to address the higher risk for depression and suicidality among diabetic patients. Additionally, several other initiatives can be considered, including: (1) early detection and treatment of depression, which would improve diabetes control and consequently, delay the development of diabetic and depression related complications; (2) the development of robust and standardized validated screening tools for diabetic patients at risk for depression and suicidality; and (3) timely design and implementation of comprehensive interventions that take into account the complex inter-relationships between depression and suicidality, so as to improve the quality of life of diabetic patients.

## 5. Conclusions

Our study found that diabetes is associated with an increased risk for depression, suicidal ideation and suicidal attempts but not completed suicide. Therefore, efforts for early detection and effective screening are urgently needed in primary care settings. Appropriate training for healthcare providers in the field of suicidality screening and depression management would help mitigate the negative impacts on a diabetic patient’s quality of life and reduce the growing burden on healthcare systems. Given the limited number of studies on this important topic, further research is needed.

## Figures and Tables

**Table 1:** Characteristics of selected depression studies.

Author &Year	Country	Study Design	Setting	Patients *N*	Age	Female %	Type of Diabetes	Outcome Reported	Evaluation of Outcome
Icks et al., 2013 [43]	Germany	Prospective cohort	Population based, mandatory residence in some cities	3663	45–75	48.6	Not specified, (included diagnosed, undiagnosed, and impaired glucose tolerance [IGT])	Depression	Center for Epidemiologic Studies-Depression Scale (CES-D) (15 items) ≥17
Cleal et al., 2017 [36]	Netherland	Prospective cohort	Population registers	3,434,420	18–59	48.9	Not specified Incident diabetes	Depression	Prescription of antidepressants
O’Connor et al., 2009 [34]	USA	Historical cohort 2 years	Patients enrolled in a health plan, services provided by primary care, family physicians and general internists	2932 with incident diabetes and 14,144 with prevalent diabetes with equal number of matched controls	≥40 Mean around 61	47.3%	Not specified but majority were type 2 International Classification of Diseases, ninth version (ICD-9)	Depression	ICD-9 ± antidepressants
Chen et al., 2013 [38]	Taiwan	Cohort	National health insurance claims and data linkage		Mean 60.1 ± 13.2	46.5	Type 2 ICD-9 Clinical Modification (CM)	Depression	ICD-9 CM
Hamer et al., 2011 [45]	UK	Prospective cohort	Community dwelling with older adults	4338	Mean 62.9 ± 9	45.2%	Not specified Self report physician diagnosis	Depression	CES-D (8 items) With a cut off ≥4
Hsu et al., 2011 [40]	Taiwan	Cohort Median follow-up 6.5 years.	Claim data, national health insurance program	14,048 diabetics and 55,608 control	≥20		Not specified (incident diabetes) ICD-9 CM	Depression	ICD-9 CM
Golden et al., 2008 [42]	USA	Prospective cohort 3.1 years.	Part of multi ethnic study of atherosclerosis	4847	45–84		Type 2, fasting blood glucose (FBG) ≥126 or on oral hypoglycemic agent (OHA) or Insulin	Depression	CED-D ≥16 or use of antidepressant medications or both
Huang et al., 2012 [39]	Taiwan	Prospective cohort 4 years.	Service claim records	200,432			Not specified ICD-9 CM	Depression	ICD-9 CM
Demakakos et al., 2014 [44]	UK	Prospective cohort	Community dwellings	4238	≥50		Not specified Self report physician diagnosis	Depression	CES-D (8 items) With a cut off ≥4
Knol et al., 2009 [37]	Netherland	Cohort	Pharmacy registry database	49,593 diabetics and 154,441 non diabetics	>40		Not specified Incident diabetes	Depression	Incident use of antidepressants
Aarts et al., 2010 [41]	Netherland	Retrospective cohort 7.7–7.9 years.	General practice patients	6140 diabetics and 18,416 control	>40–97 Mean 63.8 ± 11.2	51% among cases and 53% among control	Type 2 International Classification of Primary Care (ICPC) diagnosis based on FBG >124	Depression	ICPC code through diagnostic interview
Kivimaki et al., 2010 [35]	Finland	Cohort	Employees Record linkage	493 diabetics and 2450 control	25–65	58%	Type 2 Incident diabetes, first diagnosed as eligible to treatment	Depression	Antidepressants
Ryu et al., 2016 [46]	USA	Nested case-control	Electronic health records of primary care patients	Cases with Major Depressive Disorders (MDD) = 11,375 and equal number of controls	Median age 43	65%	Not specified At least two diagnostic codes for the condition >30 days apart	Depression	≥2 MDD-related (ICD-9-CM) diagnosis codes, ≥1 anti-depressant prescription, ≥1 mention of MDD diagnoses within inpatient or outpatient
Icks et al., 2008 [55]	Germany	Cross-sectional	Baseline data from German Heinz Nixdorf Recall study	2090 diabetic and 4595 non diabetic	45–75	50.2%	Not specified Self report physician diagnosis or medications, or FBG and random blood glucose (RBG)	Depression	CES-D short form ≥15
James et al., 2010 [47]	Nigeria	Cross-sectional	Outpatient clinic in tertiary center	200 cases and 200 control	20–64 Mean 47.1 ± 9.6	54%	Not specified, diagnosed for >1 year Based on WHO criteria	Depression	Schedule for the Clinical Assessment in Neuropsychiatry (SCAN) and Beck Depression Inventory (BDI) (21 items) ≥10
Bruce et al., 2016 [56]	Australia	Cross-sectional	Prior involvement in Brusselton Health Survey (community based study)	184 cases and 184 paired controls	Mean 70.2 ± 10.1	50%	Type 2 Self report and FBG	Depression	Patient Health Questionnaire (PHQ-9) and Brief Lifetime Depression Scale (BLDS) according to Diagnostic and Statistical Manual for Mental Disorders (DSM-VI) criteria for major and minor depression
Lin et al., 2008 [48]	17 countries	Cross-sectional	Household residing adults	42,697	Mean between 35.8–48.2	Between 47.5%–55.1%	Not specified Self report physician diagnosis or medications	Depression	Composite International Diagnostic Interview (CIDI)
Van Doreen et al., 2016 [54]	Netherland	Cross-sectional	Baseline for a population based study	862	Mean 64 ± 7	30% in diabetics and 51% in non- diabetics	Type 2 on Insulin or Oral Glucose Tolerance Test (OGTT)	Depression	Mini-International Neuropsychiatric Interview (MINI) and PHQ-9 ≥10
Foran et al., 2015 [57]	Ireland	Cross-sectional	Part of Cardiovascular Multi-morbidity in Primary Care study	283 diabetic and 283 non diabetic	>50 Mean 68 ± 9.5	41%	Type 2	Depression	Hospital Anxiety Depression Scale-Depression (HADS-D)
Chung et al., 2014 [31]	Korea	Cross-sectional	Korean National Health and Nutrition Examination Survey (KNHANES IV, V)	34,056	≥20	57.1%	Not specified Self report diagnosis, FBG ≥126, current use of anti-diabetic medications	Depression	Composite International Diagnostic Interview-Short Form (CIDI-SF)
Albertorio-Diaz et al., 2017 [58]	USA	Cross-sectional	NHANES data 2007–2012	7717	≥20		Type 1 & 2 Self report diagnosis and lab evaluation	Depression	PHQ-9, DSM-IV text revision (TR) diagnostic criteria
Berg et al., 2012 [53]	Norway	Cross-sectional	Norwegian prescription database	34,342,333	≥20	50.9%	Not specified On anti-diabetic treatment	Depression	Antidepressants
Meurs et al., 2016 [49]	Netherland	Cross-sectional	Lifeline cohort study population	90,686	18–93 Mean 45	59%	Not specified Self-reported use of anti-diabetic medication or diagnosis of diabetes	Depression	MINI
Mantyselka et al., 2011 [59]	Finland	Cross-sectional	Based on population survey, subjects	2712	45–74		Type 2 Self report diagnosis	Depression	BDI ≥10 and ≥16
Clarke et al., 2016 [50]	UK	Cross-sectional	Scottish family health study	23,690	>18	51.2%	Type 2 Self report diagnosis and medication use	Depression	Structured Clinical Interview for DSM (SCID)
Bouwman et al., 2010 [60]	Netherland	Cross-sectional		2667	40–65	46.4%	Type 2 FBG >7 mmol/L or 2hrPG 11.1 mmol/L	Depression	CES-D ≥16
Li et al., 2016 [61]	China	Cross-sectional		11,531	≥35		Not specified fasting plasma glucose (FPG) ≥7 mmol/L or previous diagnosis by a medical practitioner	Depression	PHQ-9 ≥10
Saglam et al., 2010 [62]	Turkey	Cross- sectional	Outpatient diabetes clinic	500 diabetic patients and 90 control	35–65		Type 1 & 2 Known diabetics for at least 1 yr.	Depression	BDI (21 items) >13
Kim et al., 2015 [63]	USA	Cross-sectional	NHANES 2007–2008 and 2009–2010	2266	20–79		Not specified Self report diagnosis	Depression	PHQ-9 ≥10
Islam et al., 2015 [64]	Bangladesh	Cross-sectional	Tertiary hospital attendants	591 cases and 591 control	20–60 Mean 50.4 ± 11.4	57%	Not specified Attending physician diagnosis	Depression	PHQ-9 ≥10
Wiltink et al., 2014 [65]	Germany	Cross-sectional	Gutenberg health study population	15,010	35–74 Mean 55	50.4%	Not specified Self-reported diagnosis and FBG >126 or RBG >200	Depression	PHQ-9 ≥10
Bessel et al., 2016 [51]	Brazil	Cross-sectional	Civil servants active or retired	14,447	35–74	54.1%	Not specified Self report diagnosis, medication use, HbA1c, OGTT	Depression	Clinical Interview Schedule-Revised (CIS-R) clinical interview criteria revised
Adriaanse et al., 2008 [66]	Netherland	Cross-sectional	The Hoorn study population	550	69.5 ± 6.3	49.8%	Type 2, OGTT or on treatment	Depression	CES-D ≥16
Westra et al., 2016 [67]	Netherland	Cross-sectional		527	60–87		Type 2 World Health Organization (WHO) criteria, known type 2 and using anti-diabetic medications or diet	Depression	CES-D ≥16
Lee et al., 2014 [33]	South Korea	Cross-sectional	KNHANES dataset	9159	≥40		Not specified	Depression	Single question
Ceretta et al., 2012 [32]	Brazil	Cross-sectional	Outpatients	994 cases and 2145 controls	>18		Type 2 >5 years. On insulin >1 year.	Depression and SI	MINI

**Table 2:** Data from selected studies for systematic review and meta-analysis (depression studies).

Author & Year	Outcome	Total Number of Diabetic Patients	Number of Diabetic Events	Reported Estimate (95% CI)	Adjusted Estimate (95% CI)	Adjustments
Icks et al., 2013 [43]	Depression (diagnosed diabetics)	255	18		1 (0.59–1.68)	age and sex, body mass index (BMI), myocardial infarction (MI), stroke, physical activity, education
Cleal et al., 2017 [36]	Depression	98,006	19,849			
O’Connor et al., 2009 [34]	Depression	Prevalent diabetes 14,144	1117		For subjects with low physician visits OR = 1.46 (1.19–1.8 )	Age, sex, number of primary care visits
		Incident diabetes 2932	276			
Chen et al., 2013 [38]	Depression	16,957	713		Hazard Ratio (HR) = 1.43 (1.16–1.77)	Age, sex, geographic area, urbanization statuses, and various comorbidities
Hamer et al., 2011 [45]	Depression				Odds ratio (OR) = 1.52 (1.01–2.3)	Age, baseline depressive symptoms, sex, smoking, alcohol intake, social status, C-reactive protein (CRP), Cholesterol, and BMI
Hsu et al., 2011 [40]	Depression	14,048	258	HR = 1.79 (1.54–2.07)	HR = 1.46 (1.24–1.71)	Age, sex, occupation and income and comorbidity including hypertension, stroke, hyperlipidemia and coronary artery disease
Golden et al., 2008 [42]	Depression	417	Incidence density 62/1000 for diabetic patients and 37/1000 non diabetics 60 developed depression		OR = 1.52 (1.09–2.12)	Race, ethnicity, exam site, BMI, Socio-economic status (SES), lifestyle factors, diabetes severity (dyslipidemia, hypertension (HTN), HTN medications microalbuminuria)
Huang et al., 2012 [39]	Depression	5685	331 (cumulative incidence)	Annual prevalence for diabetics 34/1000 for non-diabetics = 11/1000 Cumulative prevalence 92/1000 for diabetics and 41/1000 for non-diabetics		
Demakakos et al., 2014 [44]	Depression			OR (52–64 years.) = 2.17 (1.33–3.56) OR (>65 years.) = 0.96 (0.59–1.57)	OR (52–64 years.) = 1.83 (1.06–3.18) OR (>65 years) = 0.81 (0.48–1.37)	Age, elevated depressive symptoms at baseline, sex, marital status, education, household wealth, cardio-vascular and non cardiovascular comorbidities, BMI health behavior smoking alcohol consumption frequency and physical activity
Knol et al., 2009 [37]	Depression	49,593	7631		Relative risk (RR) = 1.71 (1.36–2.13)	Age, sex, chronic disease
Aarts et al., 2010 [41]	Depression	6140	122	HR = 1.32 (1.19–1.48)	HR = 1.26 (1.12–1.42)	Age, practice identification code and depression preceding diabetes
Kivimaki et al., 2010 [35]	Depression	493	36	OR = 2 (1.57–2.55)		Matching was based on 6 variables: age group, sex, socioeconomic position, type of employment, type of employer, and geographic area workplace
Ryu et al., 2016 [46]	Depression	237	205		OR = 2.8 (1.9–4.1)	Educational level and obesity
Icks et al., 2008 [43]	Depression	352	47		OR (male) = 0.5 (0.27–0.91) OR (female) = 1.14 (0.73–1.76)	Age, co-morbidity, depression induced medications, smoking, activity level, living without a partner, and education
James et al., 2010 [47]	Depression	200	60			
Bruce et al., 2016 [56]	Depression	184	23			
Lin et al., 2008 [48]	Depression			OR = 1.38 (1.15–1.66)		Age and gender
Van Doreen et al., 2016 [54]	Depression	253	22		OR = 1.73 (1.38–3.6)	Age, sex and education level
Foran et al., 2015 [57]	Depression	283	62			
Chung et al., 2014 [31]	Depression	3846	678	OR = 1.376 (1.258–1.504)	OR = 1.178 (1.07–1.297)	Age, sex, smoking, alcohol, education, income, physical activity, number of chronic diseases, presence of major cancer
Albertorio-Diaz et al., 2017 [58]	Depression			OR (minor) = 2.38 (1.78–3.19) OR (major) = 2.81 (1.92–4.11)	OR (minor) = 1.95 (1.39–2.74) OR (major) = 2.28 (1.45–3.57)	Effects of age, sex, race and ethnicity, education, body mass index, and poverty
Berg et al., 2012 [53]		121,392	15,511		OR = 1.53 (1.5–1.56)	Age and gender
Meurs et al., 2016 [49]	Depression	1811	90		OR = 1.39 (1.1–1.76)	Age, sex, added comorbidity and anxiety disorders
Mantyselka et al., 2011 [59]	Depression				OR (>10) = 1.35 (0.84–2.15) OR (>16) = 1.56 (0.65–3.5)	Demographic, lifestyle, and biological factors
Clarke et al., 2016 [50]	Depression	913	130			
Bouwman et al., 2010 [60]	Depression	181	38	OR = 1.86 (1.27–2.72)	OR = 1.77 (1.13–2.78)	Age, education, family history of diabetes, triglycerides, high density lipoproteins (HDL) cholesterol, total Cholesterol, hypertension, smoking and waist circumference
Li et al., 2016 [61]	Depression	529	40		OR = 1.7 (1.25–2.31)	Age, sex, and race, education level, family income, marital status, and family history of diabetes body mass index, diet score, sleep duration, current smoking, drinking status, and physical activity history of chronic disease, and any medication
Seglam et al., 2010 [62]	Depression	500	169			
Kim et al., 2015 [63]	Depression	175	41	OR = 2.24 (1.43–3.51)	OR = 1.65 (0.93–2.92)	Age, education, race/ethnicity, marital status, ratio of family income to poverty, physical activity, BMI, and waist circumference were controlled.
Islam et al., 2015 [64]	Depression	591	100		OR = 6.4 (3.4–12.3)	Education, age occupation, marital status, BMI, HTN, no of complications
Wiltink et al., 2014 [65]	Depression	1074	107			
Bessel et al., 2016 [51]	Depression	1096	63		(Prevalence ratio) PR = 1.31 (0.97–1.78)	Sex, age, race, marital status and smoking, physical activity, body mass index and waist-hip ratio.
Adriaanse et al., 2008 [66]	Depression	126	22	OR (male) = 2.04 (0.76–5.49) OR (female) = 3.18 (1.31–7.74)	OR (male) = 1.52 (0.47–4.94) OR (female) = 2.76 (1.01–7.5)	Age, low education and diabetes symptoms (hyperglycemic, cardiovascular, neuropathic pain, sensibility and ophthalmological)
Westra et al., 2016 [67]	Depression			OR = 3.04 (1.57–5.88)	OR = 1.98 (0.95–4.12)	Age, total body fat percentage, physical activity, education level, time of blood/CES-D collection, serum 25-hydroxyvitamin D, sex
Lee et al., 2014 [33]	Depression	811	152			
Ceretta et al., 2012 [32]	Depression	996	664	OR = 6.5 (5.4–7.5)	OR = 1.8 (1.7–2)	

**Table 3:** Characteristics of selected suicidality studies.

Author & Year	Country	Study Design	Setting	Patients *N*	Age	Female %	Type of Diabetes	Outcome Reported	Evaluation of Outcome
Singhal et al., 2014 [68]	England	Retrospective Cohort	Hospital day cases or inpatients	2,230,207 diabetic patient	≥10	--	Not specified (Hospital records)	Self-Harm & Suicide	Record linkage/ICD-10
Webb et al., 2012 [69]	UK	Nested Case Control	General practice research database	48,426	17–87	45.4	Not specified (ICD-9)	Self-Harm	ICD-9
Myers et al., 2013 [70]	USA	Cross Sectional	Outpatients	145	18–75	59.3	Type 2 (self- reported)	Suicide attempt	Self- reported
Radobuljac et al., 2009[25]	Slovenia	Cross Sectional		625	14–19	59	Type 1 (record data)	Suicidal ideation & attempt	Self- reported
Lee et al., 2014 [33]	Korea	Cross Sectional	KNHANES data V	8322	≥40	--	Not specified (self- reported physician diagnosis)	Suicidal ideation	Self-reported
Chung et al., 2014 [31]	Korea	Cross Sectional	KNHANES data IV, V	34,056	≥20	57	Not specified (self- reported physician diagnosis)	Suicidal ideation & attempt	CIDI-SF
Han et al., 2013 [23]	Korea	Cross Sectional	KNHANES data IV	17,065	≥20	57.6	Not specified (self- reported physician diagnosis)	Suicidal ideation	Self-reported
Igwe et al., 2013 [71]	Nigeria	Cross Sectional	Outpatient endocrinology clinic	270	18–64 mean: 51 ± 10.1	64.3	Type 1 & Type 2 at least one year after diagnosis (consultant diagnosis)	Suicidal ideation	MINI
Handley et al., 2016 [72]	Australia	Cross Sectional	Diabetes MILES national survey	3338	18–70 Mean: 51.7 (13.8)	53.8	Type 1 & Type 2 (the National Diabetes Services Scheme Register)	Suicidal ideation	PHQ-9 (item 9)
Ceretta et al., 2012 [32]	Brazil	Cross Sectional	Outpatients public health facility	994 cases and 2145 control	>18	56.6–59.2	Type 2 (self-reported)	Suicidal ideation	MINI
Sendela et al., 2015 [73]	Poland	Cross Sectional	Outpatients	477	7–18 Mean: 13.1 ± 2.7	51.3	Type 1	Suicidal ideation	CDI (Item 9)
Fuller and Sawyer, 2009 [74]	Canada	Cross Sectional	Canadian Community Health Survey (CCHS)	82,675	≥12	--	Type 1 (self-reported diagnosis and Insulin within one month of diagnosis)	Suicidal ideation	Self- reported
Batty et al., 2012 [75]	Korea	Prospective Cohort	Cancer prevention study participants	1,234,927	30–95	--	Not specified (self report physician diagnosis or medication, study detected diabetes if FBG ≥126 with no history of diabetes)	Suicide	Death Certificates
Yamauchi et al., 2016 [52]	Japan	Prospective Cohort		105,408	51.2 ± 7.9	--	Not specified (self-report of physician diagnosis or medication usage)	Suicide	Death Certificates/ICD-10
Webb et al., 2014 [76]	Sweden	Cohort	Data records	252,191 cases and 1,260,214 controls	Median 69.3 Inter quartile range (IQR) = (59.2–78.7)	44.5	Type 1 & Type 2 (diabetes register)	Suicide	Death Register
Davis et al., 2015 [77]	Australia	Cohort	Fremantle diabetes study	1413 + 5660	18–89.7 Mean: 62.3 ± 12.7	50.2	Not specified	Suicide	Death Certificate or coroner’s determination
Webb et al., 2012 [24]		Nested Case Control	Primary care longitudinal database	473 cases 17,460 controls	17–87 Median: 38	--	Not specified (ICD-9)	Suicide	ICD-10/ data linkage

**Table 4:** Data from selected suicidality studies for systematic review and meta-analysis (suicidality studies).

Author & Year	Outcome	Total Number of Diabetic Patients	Number of Diabetic Events	Reported Estimate (95% CI)	Adjusted Estimate (95% CI)	Adjustments
Singhal et al., 2014 [68]	Suicidal Attempt (SA)	2,230,207	12,433	Rate ratio (RR) = 1.6 (1.5–1.6)	--	--
	Suicide	2,230,207	626	RR = 1 (0.9–1.1)	--	--
Webb et al., 2012 [69]	Self-Harm		81	Odds ratio (OR) = 1.62 (1.28–2.06)	OR = 1.28 (1–1.64)	Clinical depression
Myers et al., 2013 [70]	S A	145	14	--	--	--
Radobuljc et al., 2009 [25]	S A	126	11	--	--	--
	Self-Harm	126	16	--	-	--
	Suicidal Ideation (SI)	126	45	--	--	--
Lee et al., 2014 [33]	SI	811	187		OR = 1.24 (0.95–1.61)	Age, sex, marital status, educational level, co-morbidities, depressive symptoms, stress
Chung et al., 2014 [31]	S A	3846	49	OR = 1.562 (1.48–2.13)	OR = 1.413 (1.02–1.96)	Age, sex, smoking, alcohol, education, income, physical activity, number of chronic diseases and presence of major cancer
	SI	3846	796	OR = 1.481 (1.36–1.61)	1.15 (1.05–1.26)	Age, sex, smoking, alcohol, education, income, physical activity, number of chronic diseases and presence of major cancer
Han et al., 2013 [23]	SI	1110	206		OR = 1.24 (1.02–1.51)	Age, sex, body mass index, household income, educational level, marital status, smoking, alcohol, and other chronic
Igwe et al., 2013 [71]	SI	270	17			
Handley et al., 2016 [72]	SI	3338	477			
Ceretta et al., 2012 [32]	SI	996	131	OR = 7.1 (5–10)	OR = 2 (1.6–2.3)	
Sendela et al., 2015 [73]	SI	477	47			
Fuller and Sawyer, 2009 [74]	SI	190	31		OR = 1.61 (1.08–2.42)	Age and sex
Batty et al., 2012 [75]	Suicide	13,452	12		Hazard ratio (HR) (male) = 2.55 (1.3–5), HR (female) = 3.64 (1.12–11.86)	Exercise, smoking status, alcohol consumption, body mass index, height, blood pressure and blood cholesterol.
Yamauchi et al., 2016 [52]	Suicide	4898	41		OR (male) = 1.2 (0.9–1.8) OR (female) = 1.5 (0.7–3)	Age at study entry, public health center area, smoking status, alcohol-drinking habits, body mass index cohabitation, employment status, hours of sleep, frequency of physical exercise, stress level and history of major physical illnesses
Webb et al., 2014 [76]	Suicide	252,191	482		RR = 3.36 (2.99–3.79)	Age, sex and country of birth
Davis et al., 2015 [77]	Suicide	1413	4		OR = 1.16 (0.38–3.51)	Age and sex
Webb et al., 2012 [24]	Suicide	892	47	OR = 1.18 (0.85–1.62)	OR = 0.9 (0.65–1.26)	Sex and age by the case-control matching with added adjustment for clinical depression.

**Table 5:** Meta-regression for depression cohort studies.

	Reference Group	Category	Coefficient	95% CI		*p*-Value	*R* ^2^
				Lower	Upper		
Depression Evaluation	Depressive Symptoms	Anti-depressants	−0.026	−0.283	0.230	0.218	0.34
		Disorders	0.126	−0.111	0.363		
Level of Adjustment ^*^	No	Full (>5)	0. 299	−0.126	0.726	0.386	0.1
		Partial (<5)	0.024	−0.162	0.212		
Female Percent			0.023	−0.002	0.048	0.07	0
Diabetes	Prevalent Diabetes	Incident Diabetes	−0.014	−0.191	0.162	0.874	0.18
Geographical location	North America	Asia	0.083	−0.155	0.032	0.111	0.36
		Scandinavian	−0.137	−0.375	0.101		
		Europe	−0.147	−0.403	0.108		

*R*^2^ reflects the amount of variability in *I*^2^ that is explained by the model. ^*^ Adjusted for <5 confounding factors was considered partial, >5 was considered full.

**Table 6:** Results for subgroup analysis for suicide and suicidal ideations.

Subgroup	*N*	Odds Ratio	95% CI		*p*-Value for Group	*p*-Value for between Groups	*p*-Value for Heterogeneity	*I*^2^ for Heterogeneity
			Lower	Upper				
**Completed Suicide**								
Sex *(unadjusted)*	0.552							
Male	2	1.536	0.78	3.027	0.215		0.068	69.96
Female	2	2.097	0.971	4.528	0.059		0.232	29.89
Sex *(adjusted)*	0.696							
Male	2	1.651	0.796	3.427	0.178		0.51	73.71
Female	2	2.059	0.895	4.733	0.089		0.21	36.34
Suicidal ideation								
Type of diabetes	0.211							
Type 1	2	1.306	0.637	2.678	0.464		0.01	84.75
Type 2 & not specified	4	2.212	1.473	3.32	<0.001		0.032	70.82
Risk of bias	0.35							
High	1	1.625	1.385	1.906	<0.001		1	0
Low	2	3.435	0.849	13.898	0.084		<0.001	98.06
Moderate	3	1.371	1.005	1.872	0.047		0.032	70.83

*N* = number of studies. *I*^2^ = percentage of variation across studies that is due to heterogeneity rather than chance.
